# High-quality PacBio draft genome sequences of 17 free-living Bradyrhizobium and four related Nitrobacteraceae strains isolated from arid soils in the Santa Catalina Mountains of Southern Arizona

**DOI:** 10.1099/acmi.0.000884.v3

**Published:** 2025-02-13

**Authors:** Melanie R. Kridler, Amanda Howe, Jimaree A. Legins, Christina Guerrero, Ryan P. Bartelme, Bridget Taylor, Paul Carini

**Affiliations:** 1 Department of Environmental Science, University of Arizona, Tucson, AZ, USA; 2 Arizona Biological and Biomedical Sciences Program, University of Arizona, Tucson, AZ 85721, USA; 3 BIO5 Institute, University of Arizona, Tucson, AZ 85721, USA

**Keywords:** nitrogen fixation, non-symbiotic, soil bacteria

## Abstract

Non-symbiotic *Bradyrhizobium* are among the most abundant and ubiquitous microbes in bulk soils globally. Despite this, most available genomic resources for *Bradyrhizobium* are derived from plant-associated strains. We present high-quality draft genomes for 17 *Bradyrhizobium* and four *Nitrobacteraceae* cultures isolated from bulk semiarid soils in Arizona, USA. The genome sizes range from 5.99 to 10.4 Mbp. Phylogenomic analysis of the 21 genomes indicates they fall into four clades. Two of the clades are nested within the *Bradyrhizobium* genus. The other two clades were associated with *Nitrobacteraceae* outgroups basal to *Bradyrhizobium*. All genomes lack genes coding for molybdenum or vanadium nitrogenases, and *nod* genes that code for proteins involved in nodulation, suggesting these isolates are free-living, non-symbiotic and do not fix dinitrogen gas. These genomes offer new resources for investigating free-living *Bradyrhizobium* lineages.

Most available *Bradyrhizobium* genome sequences are derived from symbiotic lineages that nodulate leguminous plants. Here we report the genome sequences for 17 *Bradyrhizobium* and four *Nitrobacteraceae* strains isolated from bulk soil, and their phylogenetic context. None of the genomes code for nitrogenase or nodulation genes, suggesting they are non-diazotrophic, non-symbiotic free-living soil bacteria. This work adds new genomic resources that can be used to investigate the role of non-symbiotic soil *Bradyrhizobium* in soil biogeochemistry.

## Data Summary

All genome accessions are reported in Table 1. All taxa used in the phylogenomic reconstructions and corresponding Integrated Microbial Genomics (IMG) Genome identifiers and statistics are listed in Table S1, available in the online version of this article. Genome sequences are available in both NCBI (https://www.ncbi.nlm.nih.gov/) and IMG [1].

**Table 1.** Genome summary statistics, taxonomy and isolation information

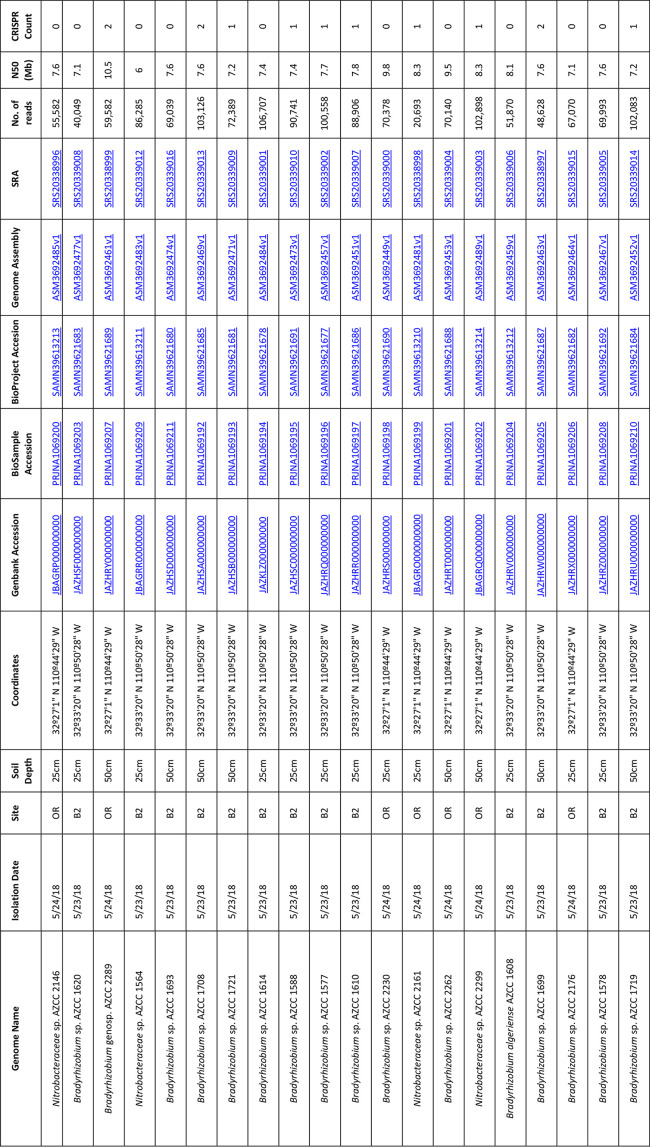 ­

## Introduction

*Bradyrhizobium* are abundant globally distributed soil microbes [2,3]. These cosmopolitan alphaproteobacteria are primarily known for their role in symbiotic nitrogen fixation with leguminous plants [4]. Yet, many, and perhaps most, soil *Bradyrhizobium* are free-living and non-symbiotic [2,5, 6]. The genomes of *Bradyrhizobium* are evolutionarily complex, and code for metabolically diverse functions [7,11]. Despite their abundance, the genomics and physiologies of non-symbiotic *Bradyrhizobium* in bulk soils are still largely unexplored [7,9]. Here, we report 21 new high-quality draft PacBio genomes of putatively non-symbiotic non-diazotrophic *Bradyrhizobium* and *Nitrobacteraceae* strains isolated from shallow subsurface soils in the Santa Catalina Mountains of Southern Arizona.

## Methods

### Culture isolation

Soil samples were collected from Catalina Mountain Critical Zone Observatory field sites in May 2018: Oracle Ridge (OR) and Biosphere 2 (B2; Table 1). Soils were kept cool on wet ice for <24 h during transport to the laboratory. We homogenised soils in sterile water with an immersion blender (10 g of 2 mm-sieved soil in 100 g water) and serially diluted them in sterile water (10-fold). Dilutions (100 µl) were plated on solid 20% Yeast Mannitol (YM) medium, consisting of (l^−1^) 0.2 g yeast extract, 2.0 g mannitol, 0.1 g K_2_HPO_4_, 0.04 g MgSO_4_, 0.02 g NaCl, 0.2 g CaCO_3_, solidified with 15 g Noble Agar (HiMedia, Kennett Square, PA). We used 20% YM instead of full strength to improve the isolation of oligotrophic microbes. Plates were incubated at 25 °C for 6 weeks. Isolated colonies from dilutions displaying isolated colonies were picked and used to inoculate arrays of deep-well 96-well plates containing 20% YM broth and incubated at 25 °C for 6 weeks.

### Culture identification

The arrayed cultures were identified and screened for *Bradyrhizobium*. The cultures were identified with a direct PCR of each well amplifying the V4–V5 region of the 16S rRNA gene using barcoded 515F and 806R primer pairs coupled with Illumina sequencing. Briefly, for each well, we amplified 16S rRNA genes in 25 µl PCR reactions containing 12.5 µl of Promega GoTaq Hot Start Colourless Master Mix; 0.5 µl of each 10 µM primer (bacterial 16S: 515F 5′-GTGCCAGCMGCCGCGGTAA-3′ and 806R 5′-GGACTACHVGGGTWTCTAAT-3′ [12]); 10.5 µl water; and 1 µl of cell culture from the arrayed 96-well plates as template DNA. Each well contained a unique barcoded 515F primer. The thermal cycler programme was 94 °C for 5 min, followed by 35 cycles (94 °C 45 s; 50 °C 60 s; 72 °C 90 s) and a final extension at 72 °C 10 min. Products were cleaned and normalised using the ThermoFisher Scientific SequalPrep Normalization Plate. Cleaned and normalised amplicons were pooled, spiked with 15% phiX and sequenced on an Illumina MiSeq using v2 500-cycle paired-end kits per the manufacturer’s instructions. Reads were processed into Operational Taxonomic Units (OTUs) and classified as described previously [13]. Wells enriched in *Bradyrhizobium* (≥75% reads matching an OTU classified as *Bradyrhizobium*) were streaked to purity on 20% YM plates. Pure cultures of *Bradyrhizobium* were verified by direct PCR using a portion of a single colony as template DNA by amplifying and Sanger sequencing the full-length 16S rRNA genes using the 27 F-1492R primer set (27F, 5′-AGAGTTTGATCMTGGCTCAG-3′; 1492R, 5′-TACCTTGTTACGACTT-3′) as described previously [13,14]. The resulting amplicons were cleaned and Sanger-sequenced by Eurofins Genomics (Louisville, KY). This resulted in 63 *Bradyrhizobium*-related cultures that are part of the Arizona Culture Collection (AZCC) housed by the Carini lab at the University of Arizona. A subset of bacterial strains from the Carini lab collection at the University of Arizona were selected for full genome sequencing at the Joint Genome Institute (JGI). These strains were chosen based on robust growth characteristics and their origins across the different sites and depths. We included several strains from the same site-depth combinations to investigate genomic differences in cohabitating populations. The species names of the AZCC genomes were based on the blast [15] best-hit of the 16S rRNA gene sequences.

### Culture scaling for genomic DNA and DNA isolation

Isolated *Bradyrhizobium* colonies were used to inoculate ‘ATCC medium: 2233 Modified Arabinose Gluconate Medium’ containing (l^−1^) 1.3 g 4-(2-hydroxyethyl)-1-piperazineethanesulfonic acid (HEPES), 1.1 g 2-(N-morpholino)ethanesulfonic acid (MES), 0.0067 g FeCl_3_·6H_2_O, 0.018 g MgSO_4_·7H_2_O, 0.013 g CaCl_2_·2H_2_O, 0.25 g Na_2_SO_4_, 0.32 g NH_4_Cl, 0.125 g Na_2_HPO_4_, 1 g l-arabinose, 1 g gluconate and 1 g yeast extract. Cultures were incubated at 25 °C with shaking at 180 r.p.m. until visually turbid (2–3 weeks). Cells were collected by centrifugation (5000×*g* for 10 min) and the supernatant was removed. DNA was extracted from pellets with a Qiagen Blood and Tissue Kit with the Gram-negative bacterial pre-treatment as recommended by the manufacturer. DNA quantity and quality were analysed using the Thermo Scientific Qubit Fluorometer and agarose gel electrophoresis, respectively.

### Genome sequencing and annotation

Genomic DNA (gDNA) was sequenced at the JGI using their in-house sequencing protocols. In brief, 1000 ng of gDNA was sheared around 10 kb using the g-TUBE (Covaris). The sheared gDNA was treated with exonuclease to remove single-stranded ends, DNA damage repair enzyme mix, end-repair/A-tailing mix and ligated with barcoded overhang adapters using SMRTbell Express Template Prep Kit 2.0 (PacBio). Libraries were pooled and purified with AMPure PB Beads (PacBio). PacBio Sequencing primer was then annealed to the SMRTbell template library and sequencing polymerase was bound to them using Sequel II Binding kit 2.0. The prepared SMRTbell template libraries were sequenced on a Pacific Biosystems' Sequel IIe sequencer using SMRT Link 10.2, 8M v1 SMRT cells and Version 2.0 sequencing chemistry with 900 min sequencing movie run times. Genomes were assembled with Flye (v. 2.8.3) [16] and annotated using the prokaryotic genome annotation pipeline (v. 6.6) [17]. Additionally, annotations are available in the comparative analysis system IMG/MER [1].

### Phylogenomic analysis

To place the new AZCC genomes in a phylogenetic context of existing *Bradyrhizobium*, we downloaded all *Bradyrhizobium* genomes available as of 1 September 2023, from the JGI IMG web portal [1] using ‘*Bradyrhizobium’* as a search query. We examined the downloaded genomes and discarded genomes that appeared to be incomplete. These excluded genomes were assembled from metagenomic data or single-cell amplified genomes. The remaining *Bradyrhizobium* dataset consisted of 489 genomes (Table S1). Additionally, we downloaded the following 21 taxa to construct the outgroup, as reported in [9]: *Bradyrhizobium* sp CCH10 C7, *Bradyrhizobium* sp CCH4 A6, *Afipia birgiae* 34632, *A. massiliensis* LC387, *A. broomeae* ATCC 49717, *A. carboxidovorans* OM5, *A. carboxidovorans* OM4, *A. carboxidovorans* OM5 DSM 1227, *Bradyrhizobium* sp U87765 SZCCT0048, *Nitrobacter winogradskyi* Nb 255, *N. vulgaris* Ab1, *N. hamburgensis* X14, *Rhodopseudomonas palustris* CGA009, *R. palustris* TIE 1, *R. palustris* DX 1. * R. palustris* BisB5, *R. palustris* HaA2, *R. palustris* BisB18, *R. palustris* BisA53, *Tardiphaga robiniae* Vaf07 and *Bradyrhizobium* sp NFR13 (Table S1).

Using the Anvi’o (v. 7.1) phylogenomics workflow [18], we investigated the phylogenomic relationships among 529 genomes encompassing the *Bradyrhizobium* genomes, new AZCC genomes reported in this study and outgroup taxa. The tree was constructed using anvi-gen-phylogenomic-tree in Anvi’o, which uses FastTree (v. 2.1.10) to reconstruct phylogenetic relationships [18,19]. The tree was based on the following concatenated ribosomal protein amino acid sequences: Ribosomal L1, L13, L14, L16, L17, L18p, L19, L2, L20, L21p, L22, L23, L27, L27A, L28, L29, L3, L32p, L35p, L4, L5, L6, L9 C, S10, S11, S13, S15, S16, S17, S19, S2, S20p, S3 C, S6, S7, S8 and S9 as identified with the Bacteria 71 HMM source in Anvi’o [18,20]. The concatenated sequences were aligned with Muscle v5 [21].

**Fig. 1.**
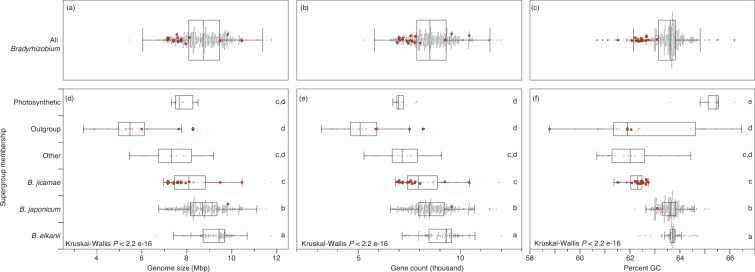
Genome statistics for 21 new AZCC isolates in the context of sequenced *Bradyrhizobium* genomes and select outgroup taxa. Points are the individual measurements of genome size, number of genes or percent guanine and cytosine (GC) for the 529 genomes included in this study. Red points are the new AZCC genomes reported here. Grey points are for existing genome sequence data. Panels (a–c) show taxa clustering within the *Bradyrhizobium* genus in Fig. 1. Panels (e–f) assign genomes to *Bradyrhizobium* and outgroup lineages as explained previously [9], see methods for details. Box plots illustrate interquartile range ± 1.5×interquartile range. The horizontal line in each box plot is the median. Outliers (>1.5 × interquartile range) are shown as points outside brackets. Kruskal-Wallis tests indicated significant variability (Kruskal–Wallis *P* < 0.05) in genome size (**d**), gene count (**e**) and per cent GC (**f**) across supergroups. Box plots sharing letters are statistically indistinguishable by Dunn’s test (Bonferroni-corrected *P*-value > 0.05).

Genomes were manually classified into *Bradyrhizobium* supergroups defined previously [9] after phylogenomic analysis. For genome statistics presented in Fig. 1, the supergroups Soil 1–3, Kakadu and taxa that did not cluster with established supergroups were combined in the ‘Other’ category.

### Nitrogenase and nodulation gene identification

We searched the genomes for genes annotated as orthologous to the KEGG Orthology numbers for nitrogenase (*nif*, *vnf*), and nodulation factors (*nod*) in IMG/ER (Table 2) [1].

**Table 2.** Distribution of KEGG Orthology (KO) genes associated with nitrogenase and nodulation in AZCC genomes

KO entry	Symbol	Name	AZCC genomes
K02586	*nifD*	Nitrogenase molybdenum-iron protein alpha chain	Absent
K02587	*nifE*	Nitrogenase molybdenum-cofactor synthesis protein NifE	Absent
K02588	*nifH*	Nitrogenase iron protein NifH	Absent
K02591	*nifK*	Nitrogenase molybdenum-iron protein beta chain	Absent
K02592	*nifN*	Nitrogenase molybdenum-iron protein NifN	Absent
K02595	*nifW*	Nitrogenase-stabilising/protective protein	Absent
K22896	*vnfD*	Vanadium-dependent nitrogenase alpha chain	Absent
K22897	*vnfK*	Vanadium-dependent nitrogenase beta chain	Absent
K22898	*vnfG*	Vanadium nitrogenase delta subunit	Absent
K22899	*vnfH*	Vanadium nitrogenase iron protein	Absent
K22903	*vnfE*	Nitrogenase vanadium-cofactor synthesis protein VnfE	Absent
K14658	*nodA*	Nodulation protein A	Absent
K14659	*nodB*	Chitooligosaccharide deacetylase	Absent
K14666	*nodC*	N-acetylglucosaminyltransferase	Absent
K14657	*nodD*	LysR family transcriptional regulator, nod-box dependent transcriptional activator	Present

## Results and discussion

### Sequencing statistics

The number of raw reads generated per genome ranged from 20 693 to 106 707 with a mean of 73 675 ± 23 136 (mean ± SD reads; Table 1). The mean contig N50 ranged from 6 to 10.5 Mbp (mean 7.8 ± 1.0 Mbp; Table 1). Each genome is assembled into a single scaffold.

### Phylogenomic placement

**Fig. 2.**
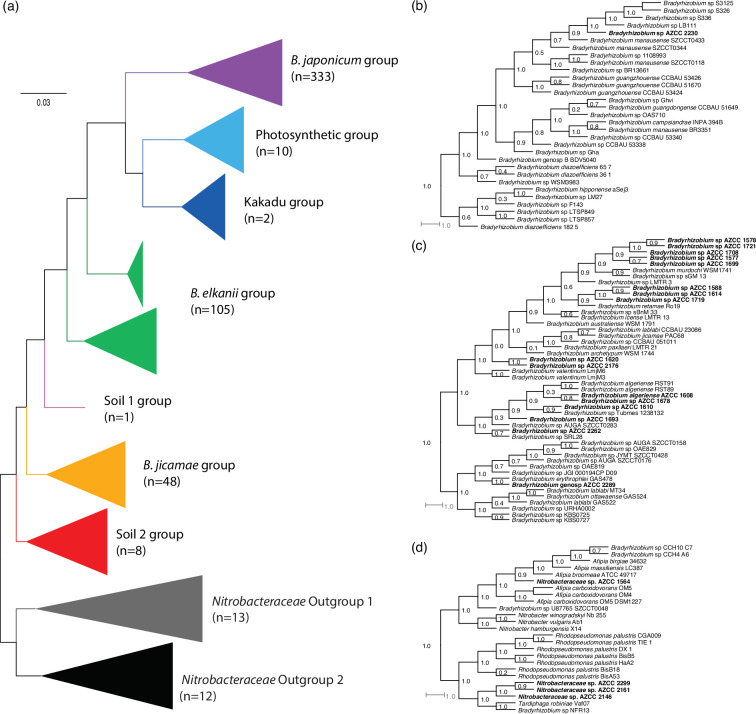
Phylogenomic relationships of the 21 AZCC isolates among sequenced *Bradyrhizobium* and *Nitrobacteraceae*. FastTree is based on 37 concatenated ribosomal protein amino acid sequences. Node labels are FastTree local support values, (a) all 529 genomes in this study. The branches are coloured and labelled by the supergroups defined in [9]. The number of genomes included in each collapsed group is listed in parentheses. Sub-trees of the *B. japonicum* supergroup (b), the *B. jicamae* supergroup (c) and the outgroup taxa (d) show the placement of the new AZCC strains (AZCC strains in bold).

Phylogenomic analysis confirmed the placement of 17 of the 21 genomes within the *Bradyrhizobium* genus (Fig. 2). *Bradyrhizobium sp*. AZCC 2230 was the sole genome that clustered within the previously described *B. japonicum* supergroup (Fig. 2a, b) [9]. Most (16 of 17) *Bradyrhizobium* strains belonged to the *B. jicamae* supergroup (Fig. 2a, c), including *Bradyrhizobium* spp., strains AZCC 1577, AZCC 1587, AZCC 1588, AZCC 1608, AZCC 1610, AZCC 1614, AZCC 620, AZCC 1678, AZCC 1693, AZCC 1699, AZCC 1708, AZCC 1719, AZCC 1721, AZCC 2176, AZCC 2262 and AZCC 2289 (Fig. 2a, c). Unexpectedly, four genomes clustered with the outgroup taxa included in our analyses (Fig. 2a, d). For example, strain AZCC 1564 clustered closely with *Afipia* isolates, while strains AZCC 2299, AZCC 2161 and AZCC 2146 clustered in a sister clade to *Rhodopseudomonas* and share an ancestor with *Tardiphaga* (Fig. 2a, d). We refer to the four strains clustering with outgroup lineages as *Nitrobacteraceae* spp. herein.

### Genome statistics

The average genome size across the new AZCC genomes was 7.87 ± 0.99 Mbp (mean ± SD) and ranged from 5.99 Mbp (AZCC 1564) to 10.4 Mbp (AZCC 2289) (Table S1). The mean genome size of the *Bradyrhizobium* AZCC genomes was 7.95 ± 0.99 Mbp (Fig. 1a). Most AZCC *Bradyrhizobium* genomes were between 7 and 8 Mbp long and are among the smallest *Bradyrhizobium* genomes reported (Fig. 1a). However, three AZCC genomes were >9 Mbp long. Two of these belonged to the *B. jicamae* supergroup (AZCC 2262 and AZCC 2289) and one to the *B. japonicum* supergroup (AZCC 2230; Fig. 1a and d). While *B. jicamae* supergroup genomes are typically significantly smaller than those in the *B. japonicum* and *B. elkanii* supergroups (Dunn’s test Bonferroni-corrected *P* < 0.05), AZCC strains 2262 and 2289 are among the largest sequenced genomes in the *B. jicamae* supergroup. Similarly, the AZCC 2230 genome is larger than the mean genome lengths in the *B. japonicum* supergroup.

As expected, the differences in *Bradyrhizobium* AZCC strain genome lengths were mirrored in the number of genes in each genome. The mean number of genes in the *Bradyrhizobium* AZCC genomes was 7807 ± 1001 genes (mean ± SD). This large degree of variability in the number of genes underscores the high variation in genetic inventories found in cohabitating free-living *Bradyrhizobium* populations. Future comparative genomic analyses among these strains are necessary to determine the functional significance of the differences in genetic inventories.

The mean per cent GC content for the AZCC *Bradyrhizobium* genomes was 62.4±0.33% (mean±SD) (Table S1) and under the median per cent, GC of all *Bradyrhizobium* genomes analysed. This result may be skewed by most new AZCC genomes belonging to the *B. jicamae* supergroup, which have a significantly lower mean per cent GC than the *B. japonicum*, *B elkanii* or photosynthetic *Bradyrhizobium* supergroups (Dunn’s test Bonferroni-corrected *P* < 0.05).

### General genome features

All genomes contained a single rRNA operon coding for a 5S, 16S and 23S rRNA. *Bradyrhizobium* is well-known to be involved in a nitrogen-fixing symbiosis with leguminous plants. None of the genomes presented here code for predicted open reading frames annotated as Mo or Va nitrogenases (*nifDEHKNW* or *vnfDKGHE*, respectively), or core nodulation genes (*nodABC*; Table 2). All genomes code for a predicted *nodD*, a transcriptional regulator (Table 2). The lack of core *nif* and *nod* machinery in a high-quality draft genome assembly strongly suggests these isolates are free-living non-symbiotic strains.

## Conclusion

These 21 new genomes nearly double the number of genomic resources available to investigate the evolution and ecology of non-diazotrophic, free-living *Bradyrhizobium* and related lineages. Additionally, they add to the growing list of genomes available from microbes isolated from semiarid soils in southern Arizona [22]. Collectively, these genomes, and the cultures they came from, can be used to experimentally identify how genetic microdiversity influences phenotypic variation across environmental stressors in the American Southwest.

## supplementary material

10.1099/acmi.0.000884.v3 Uncited Table S1.

## Abbreviations

AZCC: Arizona Culture Collection
gDNA: genomic DNA
HEPES: 4-(2-hydroxyethyl)−1-piperazineethanesulfonic acid
IMG: integrated microbial genomics
JGI: Joint Genome Institute
KO: KEGG orthology
MES: 2-(N-morpholino)ethanesulfonic acid
OR: Oracle Ridge
OTUs: operational taxonomic units
YM: yeast mannitol
